# The Process for the Formulation of the International Telehealth Position Statement for Occupational Therapy

**DOI:** 10.5195/ijt.2015.6163

**Published:** 2015-07-29

**Authors:** KAREN JACOBS, JANA CASON, ANN MCCULLOUGH

**Affiliations:** 1 BOSTON UNIVERSITY COLLEGE OF HEALTH AND REHABILITATION SCIENCES, SARGENT COLLEGE, DEPARTMENT OF OCCUPATIONAL THERAPY, BOSTON, MASSACHUSETTS, USA; 2 SPALDING UNIVERSITY, AUERBACH SCHOOL OF OCCUPATIONAL THERAPY, LOUISVILLE, KENTUCKY, USA

**Keywords:** Occupational therapy, telehealth, World Federation of Occupational Therapists

## Abstract

The World Federation of Occupational Therapists (WFOT) consists of 84 member organizations representing over 420,000 occupational therapists internationally (WFOT, 2014). In 2014, WFOT published the WFOT Telehealth Position Statement on the use of telehealth in occupational therapy. The process for the formulation of the official document involved reviewing WFOT member organizations’ telehealth position statements and data collected from a survey sent to member organizations’ delegates in April 2014. Qualitative data from 39 countries yielded factors to consider in five key areas: licensure/registration requirements, the cost of technology, privacy and security, reimbursement/payment models, and other issues (e.g., need for collaboration/transfer of knowledge, client selection, provider competencies, standard of care). The WFOT Telehealth Position Statement addressed each of these areas. The collaborative effort resulting in the development of the WFOT Telehealth Position Statement serves as a model for other international organizations.

The World Federation of Occupational Therapists (WFOT) aims to promote occupational therapy worldwide, foster international cooperation among occupational therapy associations, therapists, and other professional groups, advance the practice and standard of occupational therapy, promote ethical conduct, advance the interests of the profession, and facilitate the exchange of information (WFOT, 2011). In 2014, the WFOT published the WFOT Telehealth Position Statement. The WFOT (2014) defined telehealth as “the use of information and communication technologies (ICT) to deliver health-related services when the provider and client are in different physical locations” (p. 1). Development of the position statement involved review of existing telehealth position statements of WFOT member organizations and data collected from a telehealth survey administered by WFOT. This article describes the process used to develop the WFOT Telehealth Position Statement and includes qualitative data assimilated into the official document.

## REVIEW OF EXISTING TELEHEALTH POSITION STATEMENTS

An Internet search identified two WFOT member organizations with existing telehealth position statements: the American Occupational Therapy Association (AOTA) and the Canadian Association of Occupational Therapists (CAOT). A broader Internet search of occupational therapy associations’ websites occurred; however, no additional telehealth position statements were located. The search was restricted to sites in native English and English translation, limiting results. Terminology (e.g., telehealth, telecare) may be dependent on local interpretation and not fully capture the terms being utilized to describe the service delivery model. Also, many member organizations’ websites have content that is restricted to members only and therefore was inaccessible to researchers.

### AMERICAN OCCUPATIONAL THERAPY ASSOCIATION

The American Occupational Therapy Association’s (AOTA’s) concept of telehealth use for occupational therapy is broad and inclusive. In 2013, AOTA published *Telehealth*, a position paper wherein telehealth was defined as “the application of evaluative, consultative, preventative, and therapeutic services delivered through telecommunication and information technologies” (p. S69). This definition includes both synchronous, real-time telehealth applications (e.g., videoconferencing) and asynchronous telehealth applications (e.g., video, photographs, recorded performance data from gaming or health technologies, etc.).

In the United States, occupational therapy practitioners are using telehealth with many different populations in diverse settings, including early intervention, schools, pediatric and adult private practice, hospitals (pediatric, rehabilitation, and burn units), the Veterans Administration (VA) healthcare system, community-based mental health settings, home health, and in the workplace (AOTA, 2013; Cason & Jacobs, 2014). The AOTA telehealth position paper outlines the evidence that supports the use of telehealth in these practice settings and further points to the many benefits of telehealth for clients. The position paper affirms that the judicious use of telehealth can minimize many of the barriers to care caused by distance and other factors (AOTA, 2013).

The AOTA position paper on telehealth explicitly addresses the qualifications of the provider and ethical considerations when using telehealth technologies. Occupational therapy services provided via telehealth must be delivered by qualified professionals who abide by the same standards of care and ethical practices as for in-person delivery of occupational therapy services.

### CANADIAN ASSOCIATION OF OCCUPATIONAL THERAPISTS

The Canadian Association of Occupational Therapists (CAOT) updated its position statement on telehealth and electronic occupational therapy (e-occupational therapy) in 2011 (CAOT, 2011). The CAOT advocates for the use of tele-occupational therapy [telehealth] to improve access to occupational therapy services. The CAOT asserts that occupational therapists must maintain a high standard of care when using telehealth technologies, gain competency to enhance skill, knowledge and expertise in remote service delivery, and incorporate best practices informed by research evidence (CAOT, 2011).

The CAOT is also engaged in initiatives to educate stakeholders and advance the use of telehealth within occupational therapy, facilitate professional development activities among occupational therapists in Canada, and increase access to telehealth research to promote evidence-based practice (CAOT, 2011).

## WFOT TELEHEALTH SURVEY

In addition to reviewing existing telehealth position statements, the WFOT sent an email with a link to an online survey to all WFOT delegates in April 2014. In some cases, the WFOT delegate forwarded the survey link to the WFOT alternate or another individual within the member organization with knowledge of the topic. The survey consisted of 15 questions, including forced-choice and short answer responses (Appendix A). Forty-eight participants responded from 39 countries; 58% of participants were WFOT delegates, 19% were WFOT alternates, and 17% were representatives of their member organization, but not a WFOT delegate or WFOT alternate (Table 1).

Qualitative data (i.e., short answer responses) were particularly beneficial in identifying important issues that impact the use of telehealth in the countries represented in the survey. These issues and factors informed the ‘*Challenges and Strategies’* section of the WFOT Telehealth Position Statement. Themes that emerged included consideration for licensure/registration requirements, the cost of technology, privacy and security concerns, reimbursement requirements, and other issues including collaboration/transfer of knowledge, client selection, provider competencies, and standard of care.

## IMPORTANT ISSUES AND FACTORS IMPACTING USE OF TELEHEALTH

### LICENSURE/REGISTRATION REQUIREMENTS

Occupational therapy practitioners using telehealth technologies must abide by applicable licensure or registration requirements. To better understand licensure requirements within each represented country, survey participants were asked if occupational therapists were required to obtain a license, certificate, or registration to practice in their country. Participants were also asked to identify the body responsible for managing the license, certificate, or registration (e.g., the Ministry of Health, a national regulatory board, Occupational Therapy Union, etc.). Seventy-three percent of participants indicated that their country requires occupational therapists to obtain a license, certification, or registration in order to practice. The WFOT Telehealth Position Statement affirms that occupational therapy providers must adhere to licensure/registration requirements whether services are provided in-person or through telehealth technologies (WFOT, 2014). A WFOT’s resource document, Working as an OT in Another Country (2013), provides an overview of registration, practice, and membership requirements for many countries with representation within the WFOT.

The WFOT Telehealth Survey also asked participants to identify important factors that limit the use of telehealth within their country. Several response options were provided: no issues or factors; cost of the technology used in telehealth (e.g., computer, mobile phones, web cameras, etc.); privacy and security concerns; reimbursement and payment models for telehealth; and a short answer response option for participants to identify issues and factors not listed. Table 2 depicts the issues and factors identified by participants.

### COST OF TECHNOLOGY

The most commonly cited factor impacting the use of telehealth was the cost of telehealth technologies. Seventy-five percent of participants indicated cost of the technology used in telehealth (e.g., computer, mobile phone, web camera, etc.) was a limiting factor. While cost of technology is not specifically addressed in the document, the telehealth position statement does identify access to technology as a consideration of the client selection process.

### PRIVACY AND SECURITY CONCERNS

Thirty-three percent of participants cited privacy and security concerns as a limiting factor for telehealth adoption. One participant stated, “Careful consideration must be given to maintenance of client confidentiality with the technology tools that may be selected and used to provide telehealth services.” The WFOT Telehealth Position Statement addressed confidentiality: “Users of telehealth are obligated to employ mechanisms to ensure confidentiality for synchronous and stored client data in compliance with jurisdictional, institutional, and professional regulations and policies governing occupational therapy practice” (p. 3).

### REIMBURSEMENT REQUIREMENTS

Fifty-two percent of participants cited reimbursement and payment models for telehealth as a factor limiting the use of telehealth within their country. One participant stated:

“Occupational therapy practitioners optimize exchange of electronic mail, video, voice calls, etc. with clients within private payment systems only…socioeconomic and political, government and non-government structures have yet to be established for reimbursement and payment systems for telehealth.”

The WFOT Telehealth Position Statement requires therapists to “adhere to reimbursement requirements and accurately represent the services delivered through telehealth” (p. 3).

### OTHER ISSUES AND FACTORS

Thirty-five percent of participants indicated that ‘other issues’ limited the use of telehealth in the countries they represented. These issues were clarified in short answer responses and included the need for collaboration with local occupational therapists to increase knowledge and skills, consideration of clients’ preferences and access to telehealth technologies, and provider competencies to maintain a high standard of care.

#### COLLABORATION WITH LOCAL OCCUPATIONAL THERAPISTS

Survey participants identified the need for collaboration to transfer knowledge and create sustainable occupational therapy services, particularly in under-resourced countries in terms of availability of occupational therapy practitioners. One participant commented:

“I believe we need a statement to offer guidelines for useful and ethical use of telehealth internationally. My concerns are for guiding culturally appropriate, occupation centered, and low cost services that are directed toward growing OT in under-resourced countries. It offers tremendous opportunity for building expertise and a network of service providers and reduces the isolation and worries of some clinicians…Our great need for OT educators in under-resourced areas makes me hopeful that thoughtful use of telehealth could fill some gaps until we have in-country OT teachers to grow the profession. Another opportunity may be in the supervision of rehabilitation technician level providers.”

This sentiment was shared by another participant who stated, “It will be good for countries that have no opportunities to have enough therapists to create a network to enhance the service and uplift skills and knowledge.” Another participant specified, “I am positive about the need [of telehealth]. We can learn from the best therapists worldwide.”

The WFOT Telehealth Position Statement reaffirms the importance of collaboration, capacity building within countries, and the practice of authentic occupational therapy. To encourage collaboration with local occupational therapists, the WFOT Telehealth Position Statement advises providers to “seek opportunities to collaborate with and promote local occupational therapy providers, organizations, educational institutions, and/or associations in the interest of cohesive, relevant, and sustainable services” (p. 2). The official document further encourages authentic occupational therapy practice and “endorses practice that is client-centered and occupation-centered, and which portrays the breadth of the profession” (p. 3).

#### CLIENT SELECTION

Some of the most powerful comments related to client selection, including the clients’ perceptions of the delivery model and whether clients had access to telehealth technologies. One participant stated:

“This is an extremely important area for us, but as OTs we must also reflect on the negative effects that the use of e-based OT might have. Many of our clients, especially those who are older and those with cognitive, intellectual or psychiatric disabilities, are in a weak position when it comes to the use of computers and internet. There is a risk that the development of e-based OT creates a digital alienation for these people. OTs must make sure that this is not happening.”

Comments regarding client access and preference demonstrate the need for clinical reasoning to guide appropriate selection of clients for this service delivery model. One participant stated, “Most of the elderly population doesn’t have this kind of technology.” Another participant commented, “Consumers and providers prefer face-to-face [in-person] occupational therapy services.” Other participants echoed this sentiment, though they acknowledged potential benefits of the model within their country. For example, one participant from a country in the Middle East shared, “…current political situations make it difficult to move within the different regions of the country, however telehealth could break this barrier, but still very expensive technology and could have limited acceptance from the society.” A participant from another Middle Eastern country reported that the profession of occupational therapy is in its infancy in their country and added “at this stage we are still at the level of promoting occupational therapy services and do not think that it is appropriate to start with high technology level.”

The WFOT Telehealth Position Statement includes a statement regarding client selection:

“Therapists should use clinical reasoning to determine the appropriateness of telehealth use based on individual client situations (e.g., client’s diagnosis and impairments, nature of the occupational therapy interventions to be provided, client’s ability to access technologies). Telehealth should therefore not be used to avoid in-person services when indicated by client-specific needs, nor be used by therapists to avoid contact with clients on the basis of discrimination” (p. 2).

The client’s ability to access telehealth technologies was identified as an important consideration. Additionally, the nature of the interventions to be provided should be considered to assure that services are client-centered and represent authentic occupational therapy (e.g., occupation-based). Alienation of clients, whether intentionally (e.g., discrimination in the provision of services) or unintentionally (e.g., not managing clients’ inability to access telehealth technologies) must be avoided.

The WFOT has produced resources to support authentic, client-centered, culturally appropriate and ethical occupational therapy practice. These resources include the WFOT’s Client-centredness in Occupational Therapy (2010), Code of Ethics (2005), Definitions of Occupational Therapy from Member Organizations (2013), Diversity and Culture (2010), and Guiding Principles on Diversity and Culture (2009).

#### PROVIDER COMPETENCIES/STANDARD OF CARE

Other concerns expressed by participants related to provider competencies in the use of telehealth technology and reliability of the technology (e.g., consistent Internet speed). One participant stated, “We should ensure that high standards are achieved with activities delivered through this media.” This participant further commented, “Communication through telehealth is more challenging than with in-person service delivery; we should train our practitioners to be great communicators and savvy users of technologies.” Another participant described the need for developing providers’ competencies around telehealth:

“To engage occupational therapists fully in the implementation of telehealth, it is critical to thoroughly address concerns and reservations. Legitimate concerns, such as the unavailability of suitable devices, lapses in the reliability of technology and not being able to address the inevitable technical difficulties, frequently prevent staff from fully embracing telehealth. Therapists not only need training in how to use the technology, but also how to use it effectively to provide a successful service. They also require time to become familiar with the technologies and opportunities to practice and build their skill base. Since therapists are often called upon to support clients who encounter difficulties in using telehealth technologies, they benefit from having access to technical support, training and troubleshooting guides.”

This participant then articulated some of the challenges and specific training needs associated with developing telehealth competencies:

“The difficulties involved in translating face-to-face [in-person] services into a remote format should not be underestimated. Telehealth requires a different way of working, so therapists need opportunities to discuss concerns about maintaining the integrity and quality of services and develop protocols to guide service delivery. A good deal of contextual information is also lost when therapists are not physically present with the client. It is therefore important to develop additional skills and strategies to gather important information that is not readily accessible during a tele-consult. This type of training needs to be integrated into undergraduate training for training OTs and CPD [continuing professional development] for practicing OTs.”

The WFOT Telehealth Position Statement asserts, “Therapists must maintain professional competency, acquire competency using telehealth technologies, ensure client safety, and adhere to ethical principles of practice” (p. 3). This assertion affirms that providers must acquire professional competency around the use of telehealth and maintain a high standard of care when providing occupational therapy services through telehealth technologies.

#### TELEHEALTH INITIATIVES

Fifty-eight percent of participants reported knowledge of telehealth initiatives (e.g., political, educational, clinical) in the country they represented. These initiatives were inclusive of occupational therapy practitioners, occupational therapy students, other professions, and interdisciplinary groups. Comments about these initiatives were associated with technology, training and information exchange, and improved access to OT services:

- “Applications for [electronic tablets] and smart phones are being developed, and research projects are ongoing with partnership between engineering and health,”
- A “non-government organization (NGO) in cooperation with information technology company is developing a service for persons with physical disabilities,”
- “There is a research [study]…to use telehealth for training parents of children with cerebral palsy in their home care,”
- “Clinical supervision and health based meetings are being provided by telecommunications across the country in some locations,”
- “Occupational therapists optimize exchange of electronic mails, video, voice calls, etc. with clients,”
- “Telehealth is being used by individual practitioners as well as by therapy companies who employ occupational therapy practitioners.”

Many telehealth initiatives described in the survey aimed to improve access to occupational therapy services, particularly in rural areas. In some instances, telehealth technologies were used to facilitate communication with patients and their families or as a means to provide patient education. For example, one participant described the use of telehealth to support “communication between hospital and primary care/patient and relatives, typically on discharge from hospital.” The nature of these services included care coordination and patient/caregiver education on positioning, participation in activities of daily living, and correct use of adaptive equipment.

In many of the countries represented, telehealth initiatives were primarily being used in medicine. One participant stated, “In OT, it is not yet common, but in some interdisciplinary groups it is. Especially in the practice of specialized medicine some initiatives and practices are developing.” Responses suggested that familiarity with telehealth and telehealth technologies in other healthcare disciplines and settings may lead to increased use of this service delivery model within OT. For example, one participant shared, “I think that there will be an increased demand for these kinds of services as our clients become more and more well informed. I do think that for many clients this could be a very good way of getting in contact with OTs.”

### MODELS OF CARE

#### TELE-EVALUATION

Survey participants commented on the needs, current uses, and key considerations regarding use of telehealth to conduct occupational therapy evaluations in their respective countries. Many participants indicated that the use of telehealth for evaluation (tele-evaluation) would be beneficial to improve access to occupational therapy services, particularly for clients living in remote areas. Others alluded to the complexities of evaluation and potential limitations of telehealth technologies in this area. One participant stated:

“OTs require access to the technologies and adequate Internet to use these technologies effectively. They also require training on how to use the technologies to achieve a good evaluation…There are complexities for OTs who use a holistic or PEO [Person-Environment-Occupation] approach to evaluation in getting all the information or understandings they require using telehealth/video-call technologies. A rich understanding of the home environment is difficult to ascertain through this medium. It is also difficult to engage with a number of people and read non-verbals. Clients are also unfamiliar with this technology and therefore experience some difficulties using it effectively.”

Another important consideration for tele-evaluation is the need for further research to determine the reliability and validity of OT assessments administered remotely. This point was raised by one participant who noted, “the validity of standard evaluations when they are administered through telehealth technologies should be assessed.” The WFOT Telehealth Position Statement does not specifically address tele-evaluation or future research directions; however, the position statement does affirm that services provided through telehealth “should meet the same standards of care as services delivered in-person and comply with all jurisdictional, institutional, and professional regulations and policies governing the practice of occupational therapy (p. 1).”

#### TELE-CONSULTATION

Survey participants were asked to comment on the needs, current uses, and key considerations for tele-consultation (i.e., remote consultation between provider and client or between two providers). Many participants indicated that remote consultation could improve knowledge and skills of providers in under-resourced or remote areas. One participant commented, “This would be extremely helpful for therapists working in remote areas who need to consult with an expert colleague regarding assessment of a particular client.” Other participants described ongoing tele-consultation initiatives including consultation with parents of children with disabilities via phone and videoconferencing, clients with multiple sclerosis in the areas of energy conservation and medication management, and between therapists for the purpose of sharing expertise. In reference to ongoing tele-consultation initiatives, one participant stated, “Telehealth works well for collaboration and consultation. Therapists in rural and remote areas use this well to get advice and support from other therapists.” This participant went on to describe other applications of tele-consultation including remote consultation with builders onsite during home modification evaluations.

The ability to share knowledge and expertise via tele-consultation is a significant benefit of telehealth and is incorporated in several sections of the WFOT Telehealth Position Statement. After citing the United Nation’s (2006) *Convention on the Rights of Persons with Disabilities* proclamation for States Parties to strengthen habilitation and rehabilitation services for persons with disabilities within their communities, the WFOT position statement states:

Telehealth may improve access to services within clients’ communities and ‘strengthen and extend comprehensive habilitation and rehabilitation services’ via the transfer of knowledge and skills from remote specialists to local health-care providers through consultation and mentoring relationships. (p. 1)

Further, the WFOT’s Telehealth Position Statement’s section outlining the significance of the position to society (i.e., ‘Statement of Significance of the Position to Society’) quoted the co-produced World Health Organization and World Bank’s *World Report on Disability*, which encouraged “sharing professional expertise between countries, as well as at critical times such as in the aftermath of a disaster” (as cited in WFOT, 2014). The WFOT Telehealth Position Statement reaffirms the importance of collaboration via telehealth technologies: “Telehealth providers are encouraged to seek opportunities to collaborate with and promote local occupational therapy providers, organizations, educational institutions, and/or associations in the interest of cohesive, relevant, and sustainable services” (p. 2).

#### TELE-INTERVENTION

Survey participants described numerous examples of tele-intervention occurring within their respective countries. In one European country, a survey participant described how tele-intervention has resulted in improved access to OT services:

“There are good examples in the northern part of [European country]. In this part of our country clients may have to travel more than 300 km to be able to meet an OT for face-to-face [in person] intervention. As far as I know most of these [tele-intervention] examples are given by OTs working with hand injuries. During these interventions the OT and client use videoconferencing.”

Another participant from a country in the Middle East indicated that there were a few facilities providing tele-intervention, but stated “we need to develop this across the country.” Overall, participants’ responses validated the need for and potential benefits of tele-intervention. This perception was well articulated by one participant who commented, “Telehealth has the potential to increase equitable access at relatively low costs and efforts should be made to further implement these systems in practice.” However, concern for the potential impact of tele-intervention on the therapeutic relationship and limitations of a remote service delivery model were raised by some participants. One participant commented:

“Interventions often require hands on [methods], use of equipment, or intimate engagement. A good deal of preparation and translation of face to face therapy events into a remote delivery format is required to do this effectively using telehealth.”

The WFOT Telehealth Position Statement recognizes telehealth as an appropriate service delivery model for OT services, though clinical reasoning is needed to determine the best use of telehealth to achieve the same standards of care as services delivered in-person. The Position Statement reads:

Telehealth is an appropriate delivery model for occupational therapy services when in-person services are not possible, practical, or optimal for delivering care and/or when service delivery via telehealth is mutually acceptable to the client and provider. Telehealth can also be part of a hybrid model wherein some occupational therapy services are delivered to a client in-person, and some occupational therapy services are delivered at a distance…Occupational therapy services via telehealth should be appropriate to the individuals, groups, and cultures served, and contextualized to the occupations and interests of clients. (p. 2)

#### TELE-MONITORING

Participants commented on needs, current uses, and key considerations in the area of monitoring clients using telehealth (tele-monitoring). Participants were provided with the example of using technology to track a client’s behaviors or activity to clarify this model of care. Though the majority of participants reported no activity in the area of tele-monitoring in the country they represented, a few participants did describe ongoing tele-monitoring initiatives. One example involved the use of asynchronous data (e.g., recorded video) to remotely monitor clients’ activities and observe progress over time. This same participant indicated that these videos are sometimes shared with other specialists on the rehabilitation team to improve communication, receive feedback, and monitor treatment outcomes. A participant from a country in the European Union (EU) stated, “There is a rehabilitation project within the EU using technology at home monitored by therapists in the rehabilitation centre.” Other participants described the use of tele-monitoring for clients with multiple sclerosis (e.g., subjective registration of fatigue and activity levels using smartphone technology) and to support aging in place. Smart home technologies (e.g., Global Positioning System wearable devices, sensors and alarms) were also described as part of a telehealth delivery model (i.e., tele-monitoring) to improve safety and occupational engagement; however, it is important to note that onsite use of these devices without the remote therapeutic component does not constitute a telehealth application. Overall, participants affirmed tele-monitoring to be a valuable telehealth application in occupational therapy. This sentiment was captured in the statement of one participant:

“These technologies have some potential in assisting OTs in examining the impact and consequences of occupational engagement and assisting clients in modifying lifestyle in response to an acute or chronic health condition and promoting healthy ageing. The dedicated health monitoring devices as well as the mainstream fitness and smart devices provide opportunities to tracking and rewarding activity/occupational engagement and managing health conditions to optimise engagement.”

#### TELE-SUPERVISION

In addition to the models of care outlined above, participants were asked to comment on the needs, current uses, and key considerations in the area of supervision (tele-supervision) via telehealth technologies. Many participants commented on the need, potential benefits, and important considerations associated with tele-supervision. One participant described that:

“These technologies can assist novice and student therapists in accessing expert therapists and provide ongoing mentoring and supervision. It would reduce the travel time required to support students on placement. It would provide students and novice therapists with access to expert clinicians. The question is how best to set up a mentoring system that facilitates this.”

Though most participants did not answer this question, presumably due to having no awareness or opinion on the use of tele-supervision or unfamiliarity with the term/translation, a few participants identified tele-supervision initiatives within the countries they represented. For example, one participant commented:

“This is working very effectively in some areas but may not be the preferred choice for some occupational therapists who prefer physical face-to-face [contact] in the same environment. Having an initial face-to-face [in-person], and/or annual face-to-face [in-person] session may reduce this concern. This is an ideal form of supervision for those working in remote or isolated professional areas, or where supervision availability/skill range in local areas is limited. [Tele-supervision] relies on having the knowledge of how to use and availability of the technology.”

Another participant described personal experience with tele-supervision, “This has been done for remote sites, and I personally have done this for consultation in international fieldwork (not as the primary supervisor during the time).” Participants identified important tele-consultation considerations including adherence to regulatory and reimbursement requirements and the need to assure high standards of care when providing supervision through telehealth technologies.

## CONCLUSION

The WFOT’s Telehealth Survey demonstrated that there was a need for an official document outlining the WFOT’s position on the use of telehealth in occupational therapy. Input from representatives of member organizations and review of existing occupational therapy telehealth position papers/statements informed the WFOT’s Position Statement on Telehealth, which addressed important practice considerations including licensure/registration requirements, need for collaboration/transfer of knowledge, client selection, provider competencies, and standard of care among other areas. Through the process of reviewing available occupational therapy telehealth position papers and statements and engaging WFOT member organizations, the WFOT created a valuable resource that will enhance the integration of telehealth in occupational therapy and established a process that may serve as a model for other international organizations.

## Figures and Tables

**Table 1:** Countries Represented in the WFOT Telehealth Survey

Countries Represented in the WFOT Telehealth Survey
Argentina
Australia
Austria
Belgium
Canada
Chile
Columbia
Croatia
Denmark
Georgia
Germany
Greece
Iran
Ireland
Israel
Jordan
Kenya
Korea (Republic of)
Latvia
Luxembourg
Malaysia
Malta
Netherlands
New Zealand
Norway
Pakistan
Palestine
Philippines
Portugal
Romania
South Africa
Sri Lanka
Sweden
Switzerland
Thailand
Tunisia
Turkey
United States of America
Venezuela

**Table 2:**
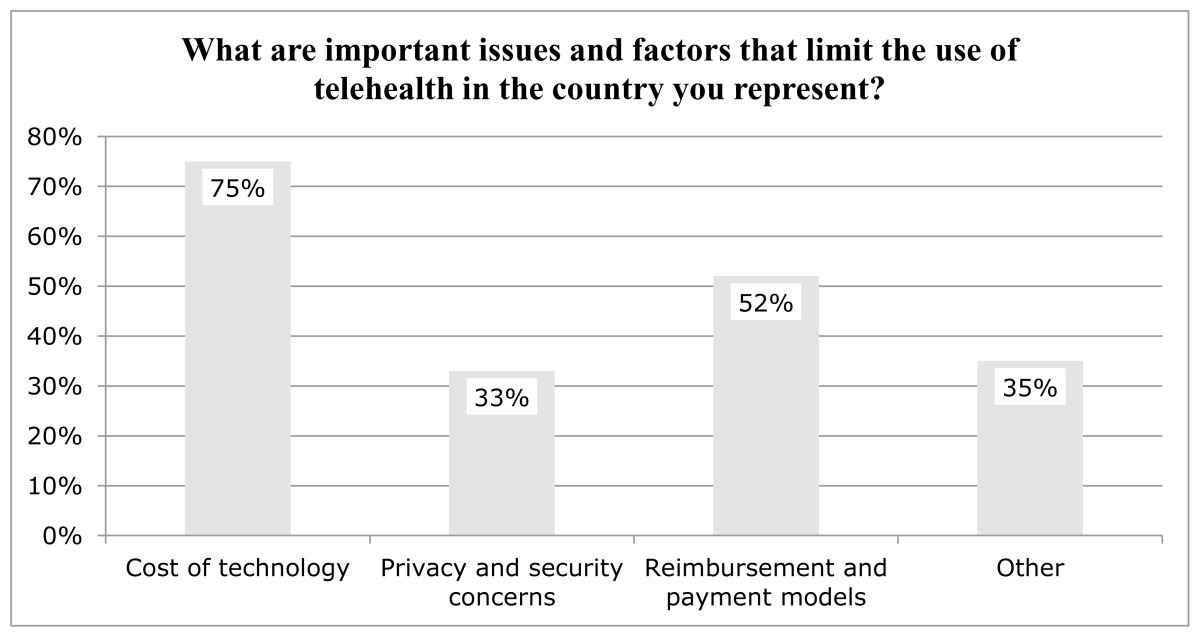
Issues and Factors that Limit the Use of Telehealth

## APPENDIX A

### Survey Items

Q1: Responses will only be recorded when you press “DONE” at the end of the survey. Submission of the survey implies consent to have your responses used for review purposes. Member Organisations that respond or do not respond to the survey will be included in the results and may be used by WFOT for publications and presentations.

Answer Choices:

- I consent to taking part in the survey.
- I do not consent to taking part in the survey.

Q2: You will be responding on behalf of a WFOT Member Organisation, please select from the list the organization you represent:

Answer Choices:

- Drop down list with the 84 WFOT Member Organisations listed

Q3: Please indicate your role in the country you represent:

Answer Choices:

- WFOT Delegate
- WFOT Alternate
- Member Organisation representative (other than Delegate or Alternate)
- Other (please specify)

Q4: Does the country that you represent require occupational therapists to obtain a license, certificate or registration in order to practice?

Answer Choices:

- Yes
- No

Q5: Please indicate who administers the license/certificate/registration (examples: the Ministry of Health, National Regulatory board, Occupational Therapy Union)

Answer Choice:

- Free response answer

Q6: What are important issues and factors that limit the use of telehealth in the country you represent?

Answer Choices:

- No issues or factors
- Cost of the technology used in telehealth, e.g. computer, mobile phones, web cameras, etc.
- Privacy and security concerns
- Reimbursement and payment models for telehealth
- Other (please specify)

Q7: Are there any telehealth initiatives (e.g. political, educational, clinical) that you are aware of in the country you represent? This could be by occupational therapy practitioners, occupational therapy students, other professionals or interdisciplinary groups.

Answer Choices:

- No
- Yes (please share any comments you have about these efforts)

Q8: Does the country that you represent have a position statement or policy on the use of telehealth technologies by occupational therapy practitioners?

Answer Choices:

- No
- Yes (please describe how to access the Telehealth statements/guideline/position paper or email us a copy)

Q9: Please comment on the needs, current uses, and key consideration in the area of evaluation of clients using telehealth

Answer Choice:

- Free response answers

Q10: Please comment on the needs, current uses, and key considerations in the area of consultation using telehealth. For example, the consulting of provider to provider, or provider to client using technology.

Answer Choice:

- Free response answers

Q11: Please comment on the needs, current uses, or key considerations in the area of client intervention using telehealth. For example, the use of a virtual intervention or treatment session.

Answer Choice:

- Free response answers

Q12: Please comment on the needs, current uses, and key consideration in the area of monitoring of clients using telehealth. For example, using technology to track a client’s behaviors or activity.

Answer Choice:

- Free response answers

Q13: Please comment on the needs, current uses, and key consideration in the area of supervision of occupational therapists using telehealth. For example, the use of videoconferencing to oversee occupational therapy students or occupational therapists.

Answer Choice:

- Free response answers

Q14: Please comment on any research you are aware of that is being conducted on telehealth in the country you represent.

Answer Choice:

- Free response answers

Q15: Please share any further information for the WFOT to consider in drafting a position statement in the use of telehealth in occupational therapy.

Answer Choice:

- Free response answers
